# Pegvisomant in acromegaly: an update

**DOI:** 10.1007/s40618-017-0614-1

**Published:** 2017-02-07

**Authors:** A. Giustina, G. Arnaldi, F. Bogazzi, S. Cannavò, A. Colao, L. De Marinis, E. De Menis, E. Degli Uberti, F. Giorgino, S. Grottoli, A. G. Lania, P. Maffei, R. Pivonello, E. Ghigo

**Affiliations:** 1 Chair of Endocrinology, Vita-Salute San Raffaele University, Milano, Italy; 2 Clinic of Endocrinology and Metabolism Disease, Ospedali Riuniti di Ancona, Ancona, Italy; 3 Department of Clinical and Experimental Medicine, University of Pisa, Pisa, Italy; 4 Department of Clinical and Experimental Medicine, University of Messina, Messina, Italy; 5 Department of Clinical Medicine and Surgery, University of Naples Federico II, Naples, Italy; 6 Pituitary Unit, Catholic University School of Medicine, Rome, Italy; 7 Department of Internal Medicine, General Hospital, Montebelluna (TV), Italy; 8 Section of Endocrinology and Internal Medicine, Department of Medical Sciences, University of Ferrara, Ferrara, Italy; 9 Department of Emergency and Organ Transplantation, Section of Internal Medicine, Endocrinology, Andrology and Metabolic Diseases, University of Bari Aldo Moro, Bari, Italy; 10 Endocrinology, Diabetology and Metabolism, AOU Città della Salute e della Scienza of Turin, Turin, Italy; 11 Endocrinology Unit, Department of Biomedical Sciences, Humanitas University and Humanitas Research Hospital, Rozzano, Italy; 12 Department of Medicine (DIMED), 3rd Medical Clinic, Azienda Ospedaliera Padova, Padova, Italy; 13 Department of Clinical and Surgery Medicine, Endocrinology and Metabolism, University of Naples, Naples, Italy; 14 Department of Medical Sciences, School of Medicine, University of Turin, Turin, Italy

**Keywords:** Acromegaly, Pegvisomant, Review, SRL resistance, Metabolic effects, IGF-I

## Abstract

**Background:**

In 2007, we published an opinion document to review the role of pegvisomant (PEG) in the treatment of acromegaly. Since then, new evidence emerged on the biochemical and clinical effects of PEG and on its long-term efficacy and safety.

**Aim:**

We here reviewed the emerging aspects of the use of PEG in clinical practice in the light of the most recent literature.

**Results:**

The clinical use of PEG is still suboptimal, considering that it remains the most powerful tool to control IGF-I in acromegaly allowing to obtain, with a pharmacological treatment, the most important clinical effects in terms of signs and symptoms, quality of life and comorbidities. The number of patients with acromegaly exposed to PEG worldwide has become quite elevated and the prolonged follow-up allows now to deal quite satisfactorily with many clinical issues including major safety issues, such as the concerns about possible tumour (re)growth under PEG. The positive or neutral impact of PEG on glucose metabolism has been highlighted, and the clinical experience, although limited, with sleep apnoea and pregnancy has been reviewed. Finally, the current concept of somatostatin receptor ligands (SRL) resistance has been addressed, in order to better define the acromegaly patients to whom the PEG option may be offered.

**Conclusions:**

PEG increasingly appears to be an effective and safe medical option for many patients not controlled by SRL but its use still needs to be optimized.

## Introduction

Acromegaly is a rare chronic disease caused by growth hormone (GH) hypersecretion, which is due in more than 95% of cases to a GH-secreting pituitary adenoma. This hypersecretion of GH and the associated elevated IGF-I levels result in increased morbidity and mortality. Surgery is in general the first treatment modality, but it is not always successful, as the majority of the patients have a macroadenoma which is often invasive. Medical treatments include somatostatin receptor ligands (SRL), dopamine agonists, and pegvisomant (PEG), a genetically engineered GH-receptor antagonist [1–3]. Octreotide and lanreotide, especially in their long-acting formulations, are the most widely used SRL to treat acromegaly; they have shown to normalize GH and IGF-I levels in about half of patients and reduce tumor mass in a considerable proportion of patients [4, 5]. More recently, the multireceptor-targeted SRL pasireotide has shown, in Phase III clinical trials, to achieve better biochemical disease control than octreotide LAR, but to be associated with a greater frequency and degree of hyperglycemia and diabetes mellitus [6, 7].

PEG has a unique mechanism of action, antagonizing endogenous GH at the GH receptor level and thereby lowering IGF-I production and ameliorating the clinical features associated with acromegaly. In the case of PEG, serum IGF-I is the sole biomarker of drug efficacy [1, 2, 8]. PEG has been shown to control acromegaly in 60–90% of patients across several clinical trials [9–18]. In Europe, PEG was approved in 2002 for patients treated with neurosurgery and/or radiotherapy and not controlled by other medical treatments, who may represent up to 40% of acromegalic patients [19], and since then it was shown to effectively control IGF-I secretion in many of these subjects [20].

In 2007, we published an opinion document to review the role of PEG in the treatment of acromegaly [21]. Since then, new evidence emerged on the biochemical and clinical effects of PEG and on its long-term efficacy and safety. Therefore, we here propose an updated review focusing on the emerging aspects of the use of PEG in clinical practice in the light of the most recent literature. *Although combination therapy is currently not included in the PEG label, this review will consider also articles where PEG was administered in combination with SRL, since this approach is sometimes used in the clinical practice*.

## Biochemical outcome

The most recent guidelines recommend IGF-I measurement as the effective biomarker of PEG efficacy [1, 2]. However, the lack of a shared standardization, the use of different antibodies, and interferences due to binding proteins hampers the comparison of the results obtained with different commercial IGF-I assays [22]. In addition, population and age-specific normal ranges are rarely provided even by laboratories of secondary and tertiary referral centers, despite this had been strongly recommended [2, 23]. For these reasons, we believe that it is crucial to monitor disease activity in each patient using the same IGF-I assay throughout the treatment period.

PEG normalizes IGF-I levels in the majority of patients. In pivotal trials, IGF-I normalization was achieved in up to 95% of patients resistant to SRL treatment [9, 24, 25]. In more recent studies, in the real life setting, the rate of disease control acromegaly was lower, not exceeding 65–70% [13, 26–28]. This lower than expected efficacy, observed especially in retrospective studies, could be explained by an inadequate dose titration of the drug, poor compliance to daily injections, selection bias of patients recruited in clinical trials in terms of disease activity and drug responsiveness, or technical problems related to IGF-I assay, while a true “biochemical resistance” to PEG cannot be ruled out yet [29]. Recently, a comparison between secondary and tertiary referral centers, identified on the basis of the number of acromegalic patients treated with PEG, failed to demonstrate significant differences in IGF-I normalization rates [30]. According to a recent study, the response to PEG as second line medical therapy can be predicted on the basis of patient’s age, BMI, and baseline IGF-I levels, but not of subjective symptoms [28]. Accordingly, PEG starting dose should be higher and dose titration more rapid in younger patients, in the obese subjects and in those with a worse endocrine profile [31, 32]. Previous studies also showed that IGF-I normalization requires lower doses in male than in female patients, and in irradiated compared to non-irradiated cases [17, 26, 33]. On the other hand, an appropriate PEG dose titration up to the maximum allowed dosage was shown to normalize IGF-I levels in up to 90% of cases, even in the real life setting [34]. It may be concluded that, although the maximum authorized dosage is 30 mg daily, there is evidence that some patients may respond to doses of 40 mg daily and it can be hypothesized that selected cases may respond to even higher doses [34].

PEG exhibits a favorable effect on the glycemic profile and can be useful in acromegalic patients with diabetes mellitus [21]. However, the rate of IGF-I normalization is reduced in patients with diabetes mellitus (64% in diabetic vs 75% in non-diabetic patients), and higher PEG doses are necessary to control the disease [35]. Possibly, it can be speculated that hyperinsulinism may cause an increased expression of hepatic GH receptors, which in turn may need higher doses of PEG to be saturated [36].

Data concerning the role of the GH-receptor truncated variant (d3GHR) in the biochemical response to PEG are controversial. Initial studies reported a better efficacy of the drug in patients with d3GHR as compared with those with full-length receptor [37, 38]. However, these data were not confirmed by subsequent studies, both using PEG alone and in combination with SRL [19, 39]. A significant role for d3GHR in the biochemical monitoring of acromegaly after surgery is also debated [40–42].

Serum GH levels are not a useful marker of PEG efficacy and should not be measured during the treatment, because of the cross-reactivity of the drug with endogenous GH in commercial GH assays. However, it has been speculated that a sudden GH increase during PEG treatment could indicate pituitary tumor growth [43].

## Global clinical effects

### Effect on pituitary tumor size

Much attention has been paid to a possible increase in tumor size associated with PEG treatment, but also sometimes under SRL treatment, even though no causal relationship has been established [44]; however, since tumor enlargements are detected increasingly often thanks to modern techniques, higher consideration should be given to the actual clinical relevance of such enlargements.

Data on tumor volume outcome in patients treated with PEG alone or in combination with SRL are discordant and widely discussed. Some long-term evaluations reported an increased tumor size during PEG in 5–7% of patients [45]. In the Italian ACROSTUDY patients, according to MRI at local centers (data available for 249 patients), a decrease in tumor volume was reported at least once in 13.7%, an increase in 8.8%, and both increase and decrease in 6.4%, in different treatment phases. Bianchi et al. [46] observed that none of 35 patients under PEG alone showed significant tumor growth, whereas in one case, MRI documented progressive shrinkage of the adenoma, which was no longer detectable after 6 years of treatment. In the same study, among the 27 patients treated with PEG in combination with SRL, a significant growth (>25%) of the residual adenoma tissue occurred in one case. This patient had from the beginning a very aggressive disease that was difficult if not impossible to control, and, when the tumor enlargement was noted, was receiving PEG 40 mg/day plus lanreotide ATG 120 mg every 4 weeks.

Tumor growth may be observed during the first year of treatment with PEG and may prevalently reflect the disease natural history or the consequence of SRL discontinuation (rebound phenomenon) [47]. Absence of previous irradiation and shorter duration of SRL therapy before PEG were associated with increased risk of tumor growth [45]. Changes in tumor size seem not to correlate with IGF-I levels [48]. Optimal use of PEG with respect to tumor size should take into account the history of the adenoma in terms of aggressiveness and invasiveness, previous tumor response to treatments including SRL and tumor volume at treatment start [45].

Despite the few reports of increase in tumor size during PEG treatment, there is no clear evidence that PEG may directly promote tumor growth [47]. Due to the interpersonal variability in pituitary, MRI reading by a single neuroradiologist of all available images before and during PEG therapy is recommended to avoid misinterpretations.

### Effect on clinical symptoms and signs, and on quality of life

Recent improvement in the medical treatment of acromegaly has resulted in better biochemical disease control [49–51]. Normalization of both GH and IGF-I levels was demonstrated to restore normal life expectancy in acromegaly patients. However, biochemical control does not necessarily relieve all symptoms [52, 53]. To quantify the symptoms and the perceived health in patients with acromegaly, the Patient-Assessed Acromegaly Symptom Questionnaire (PASQ) and the Acromegaly Quality of Life Questionnaire (AcroQoL) have been developed [52–54]. In particular, the PASQ score is a disease-specific tool that rates five features of acromegaly: headache, excessive perspiration, arthralgia, fatigue, and soft tissue swelling [52]. On the other hand, the AcroQoL questionnaire contains 22 items divided into two scales, one evaluating physical features and the other assessing psychological aspects [53, 54]. The use of these questionnaires may improve clinical evaluation of therapeutic interventions, and identification of patients who possibly require further treatment. Both treatments with SRL and PEG showed to be effective in reducing acromegaly signs’ and symptoms’ total scores, and improving health-related quality of life (QoL) [55–57]. However, in patients with more severe disease (IGF-I ≥ 2xULN), a trend toward greater improvements of PASQ and AcroQoL scores was observed with PEG compared with octreotide LAR [57]. On the other hand, a lower convenience score was observed in patients treated with PEG and evaluated by the Treatment Satisfaction Questionnaire for Medication (TSQM), that may be explained by daily injections [58].

PEG therapy was widely demonstrated to be effective and safe in acromegalic patients, improving clinical symptoms and systemic complications [24, 26, 27]. In fact, Trainer et al. reported significant decreases in mean patient-assessed symptom scores, including soft tissue swelling, degree of perspiration and fatigue, as well as in the mean sum scores of all five PASQ items, in patients treated with 15 or 20 mg of PEG per day [24]. Subsequently, many other studies [25, 59–61] have demonstrated a significant improvement in acromegaly-related QoL, and clinical signs and symptoms in patients treated with PEG in monotherapy or in combination with SRL. However, the improvement was observed in some but not all measures of perceived health or QoL scores. In particular, pain syndrome, joint complaints, and persistent headache seem to persist regardless of type of therapy and/or biochemical control [24, 52]. A possible explanation for the different responsivity of symptoms to PEG therapy may lie in the nature of symptoms themselves [52]. Skin manifestations, such as perspiration, soft tissue swelling or numbness, caused by an increase in extracellular volume, edema, dermal glycosaminoglycan accumulation or increased vasoconstriction, are usually rapidly alleviated when GH and IGF-I levels are controlled [52, 62]. Conversely, joint pain may rather reflect chronic and irreversible changes [63, 64], so that biochemical control per se may not be sufficient to alleviate them, whereas headache may better respond to centrally active drugs like SRL [65]. For these reasons, additional pain treatment may be needed to relieve these symptoms.

Overall, 11 independent studies [9, 14, 24–26, 31, 33, 52, 59, 60, 66] specifically focused on headache in acromegalic patients on PEG either as monotherapy or in combination with SRL. Treatment with PEG has been generally reported to negatively impact on headache in acromegalic patients, although not significantly. Actually, the headache score at PASQ was found slightly and not significantly impaired in several studies [31, 52, 59]. In another study, the headache score in patients on PEG monotherapy was not significantly higher than that of patients on combined SRL and PEG treatment [60]. In patients receiving PEG monotherapy, headache has been described in a widely variable percentage, ranging from 2% [14, 26] to 40.7% [66]. However, the prevalence of severe headache leading to definitive treatment discontinuation ranged from 1.2% [24] to 28.6% [31]. Noteworthy, the association between worsening of headache score and increase in tumor size while on PEG therapy is still to be fully elucidated. Two different studies have reported discordant results, with a correlation between worsening of headache and increase in tumor size in the first study [66] but not in the second one [33]. Curiously, in a study [25] comparing long-term therapy with PEG alone, PEG combined with octreotide LAR, and octreotide LAR alone, headache was reported only in patients on octreotide LAR monotherapy and was related to the disease state.

Taken altogether, these data suggest that the normalization of IGF-I may induce an improvement in most acromegaly-related symptoms and QoL. However, normalization of clinical picture from the patient’s perspective not always is obtained, and further studies are needed to investigate the clinical impact of treatment with PEG alone and combined with SRL in larger series of patients.

## Effect on comorbidities

### Glucose and lipid metabolism

In acromegaly, abnormal glucose tolerance, hyperinsulinemia, and diabetes mellitus are frequently observed; these abnormalities contribute to the increase in cardiovascular morbidity and mortality of the disease. In particular, GH excess increases glucose production through the hyperstimulation of lipolysis, which provides free fatty acids (FFAs) and glycerol as metabolic substrates, and inhibits insulin-induced suppression of hepatic gluconeogenesis [67, 68]. All these effects have a negative impact on insulin action, reducing insulin sensitivity [68]. Treatment of acromegaly may influence glucose metabolism in different ways. It is known that SRL inhibit insulin secretion, inducing an unpredictable impact on glucose homeostasis [68]. This effect may potentially impair glucose homeostasis in acromegaly, especially in patients with pre-existing glucose intolerance or type 2 diabetes. Indeed, there is evidence that currently available SRL, lanreotide autogel (ATG), and octreotide long-acting release (LAR) may have an overall marginal clinical impact on glucose homeostasis in this setting [69], even when used at high doses [70]. However sometimes, a clinically significant deleterious glycometabolic effect may be observed in some patients treated with conventional SRL, especially when acromegaly is not biochemically controlled by the treatment and high doses of analogs are administered (70).

Pasireotide, a new somatostatin receptor ligand that has been recently approved for the treatment of acromegaly, binds with higher affinity than octreotide and lanreotide to somatostatin receptor subtype 5, which is highly expressed in pancreatic β cells and modulates insulin secretion [71]. Studies performed in healthy volunteers showed that pasireotide inhibited insulin secretion and incretin response, with minimal inhibition of glucagon secretion and no impact on insulin sensitivity [72]. In acromegaly, pasireotide was shown to cause hyperglycemia and diabetes more frequently than octreotide LAR, even in patients with better-controlled acromegaly [73]. Conversely, PEG therapy has been reported to have more favorable effects on glucose homeostasis than conventional SRL [29, 74] by improving insulin sensitivity and endogenous glucose production rate, reflecting predominantly hepatic glucose production, while decreasing overnight FFA levels [75].

Several studies have demonstrated that PEG, used as monotherapy, induces a significant decrease in fasting glucose levels also in patients with diagnosed diabetes mellitus and impaired glucose tolerance (IGT); there is also evidence of an improvement in glucose tolerance and a decrease in HbA1c levels [76, 77]. A positive impact of PEG on peripheral insulin sensitivity has also been demonstrated, by a significant improvement at the short insulin tolerance test [14], and at the hyperinsulinemic euglycemic clamp [78, 79] but not when using the homeostatic model assessment. Conversely, the effect of PEG on FFAs is still controversial. Recent studies failed to demonstrate a reduction in FFAs, whereas previous papers [79, 80] reported a significant reduction in serum FFAs after successful PEG treatment, explaining, at least in part, the improvements in hepatic insulin sensitivity. However, it is important to note that a substantial proportion of patients in those studies were completely or partially resistant to SRL; therefore, improvement in glucose metabolism might be rather due to better biochemical control and to the reverted inhibitory effect of the SRL on insulin secretion.

These findings suggest that in patients resistant to SRL with uncontrolled diabetes mellitus or worsening hyperglycemia, PEG monotherapy may be the first medical option, since it may allow to reach the combined objective of improving both biochemical control and glucose metabolism.

Variable results were observed on lipid metabolism after PEG. An increase in total and LDL cholesterol (LDLc), with unchanged triglyceride levels and a significant decline in lipoprotein (a) levels was observed [51]. Small, uncontrolled studies reported no change of lipid profile during PEG therapy. Kuhn et al. found that long-term treatment with PEG seems to be associated with minimal increase in plasma LDLc [81].

Data on changes in BMI during treatment with PEG are scarce and conflicting: some studies have shown an increase in BMI [82, 83] while others showed no change [84, 85]. Overall, however, the data suggest an increase in fat mass [86], particularly intra-abdominal fat mass [87], and also a decrease in lean body mass and extracellular water, which might explain the neutral effect of PEG on BMI in some studies [81].

### Cardio-respiratory system

In acromegalic patients highly resistant to SRL, long-term (at least 18 months) treatment with PEG induced a significant reduction of cardiac mass. Particularly, in a retrospective study, Kuhn et al. evaluated, by echocardiography, the long-term (up to 10 years) impact on cardiovascular comorbidity in a series of 42 acromegalic patients treated with PEG alone (19 cases) or in combination with SRL and/or cabergoline (23 cases). The authors showed that 20 ± 16 months after PEG introduction, left ventricular ejection fraction (LVEF) improved significantly in patients with systolic dysfunction and decreased or normalized in acromegalic patients with a hyperkinetic syndrome [80]. Moreover, a decrease in LV mass index was reported in patients with most severe LV hypertrophy. Treatment with PEG also exerted beneficial effects on rhythm disorders and hyperkinetic syndrome, carotid arteries wall thickness, and blood pressure, particularly in terms of diastolic values, in hypertensive patients [51].

Respiratory disorders are frequent complications in patients with acromegaly, with a potential impact on both morbidity and mortality [88]. The most frequent respiratory complication of acromegaly is the obstructive sleep apnoea syndrome (OSAS) with a prevalence up to 80% of patients at diagnosis [88–90]. The pathogenesis of the OSAS in acromegaly is complex, including systemic as well as local effects of GH and IGF-I excess, such as alterations in the facial bone structure, soft tissue swelling of the upper airways, and macroglossia. The relationship between activity of the disease and severity of OSAS in acromegaly is controversial: studies have shown either a positive or no correlation between serum GH and IGF-I levels and indices of sleep apnoea [90–93]. The impact of medical therapies for acromegaly on the prevalence and the severity of OSAS is not clear, although some studies reported an improvement of OSAS severity after biochemical control of disease was achieved by SRL [94, 95].

The effect of PEG on respiratory complications of acromegaly has been evaluated in two studies including a small number of patients [81, 96]. In one study, twelve subjects with uncontrolled acromegaly under SRL were evaluated using polysomnography and MRI of the tongue before and 6 months after the introduction of PEG [96]. In that study, the achievement of biochemical control obtained by PEG was associated with an overall improvement of the indices of apnoea [i.e., reduction of the severity of sleep apnoea and of the apnoea-hypopnea index (AHI)] and with a significant shrinkage of the tongue volume [96]. In addition, the authors reported a correlation between IGF-I levels and tongue volume, whereas AHI improvement did not correlate with the decrease in IGF-I levels, nor with BMI, BMI-adjusted tongue volume or age. The other assessment of the effect of PEG on sleep apnoea was conducted in a subset of twelve acromegalic subjects included in a retrospective single-center study on the effects of the treatment with the GH-antagonist, alone or associated with other treatments, on the systemic comorbidities of acromegaly [81]. The results of this study were consistent with those of the previous study, showing a significant improvement of AHI and a reduction of the severity of the sleep apnoea after the introduction of PEG and the improvement of the biochemical control of acromegaly.

Overall, the results of these two studies suggest a positive impact of PEG therapy on the severity of apnoea; larger prospective studies are needed to evaluate the impact of PEG on the incidence of OSAS.

### Bone

In acromegaly, there is an increase in the risk of vertebral fractures [97], which is not necessarily associated with reduced bone mass [98], but rather with an increased bone turnover [99] that was normalized after 6 months of PEG treatment [100]. Long-term treatment with PEG also induced a significant increase of bone mineral density in active acromegaly [101]. Although PEG use was weakly associated with an increased rate of fractures, this has been attributed to global increased severity of the disease in treated patients [102].

## Safety

### Liver test abnormalities

A generally mild and transient liver transaminase levels elevation (LTLE) was reported in about 5–8% of patients in surveillance studies, the highest percentages being generally [45, 48] but not always [46] observed when PEG was combined with SRL. Different risk factors for developing LTLE have been hypothesized, such as type 2 diabetes mellitus, the common polymorphism (UGT1A1) of Gilbert’s syndrome, and male sex [47]. Of the 341 acromegaly patients included in the Italian ACROSTUDY, liver test abnormalities (>3 × ULN of ALT and AST, >1 × ULN of ALP, bilirubin or GGT) were observed in 27 patients. In three patients (0.9%), a clinically significant increase of transaminases (>5 × ULN of ALT or AST) was reported after 3 months of PEG therapy, but levels normalized spontaneously or after PEG withdrawal (data available for 320 cases) [27].

### Lipodystrophy

Injection-site reactions, reported with a frequency up to 11%, were generally mild, erythematous, self-limited, and did not require treatment [26]. Lipodystrophy, a localized disorder of adipose tissue resulting in depressed skin areas (lipoatrophy) or areas of fat overgrowth (lipohypertrophy), has been observed during PEG therapy due to local GH lipolysis inhibition [18, 26, 103–106].

In 1288 patients of the ACROSTUDY, lipodystrophy was reported as a minor side effect with a very low prevalence (1.4%) [26]. However, a recent multicentre retrospective study involving 19 Spanish centers [18] reported lipodystrophy development in 15% of patients (15/97), mostly females. Fourteen patients had lipohypertrophy and one lipoatrophy. When all possible treatment associations were analyzed, only the triple association of SRL, cabergoline, and PEG was related to a higher incidence of lipodystrophy (42 vs 11%, *p* = 0.018). Interestingly, lipodystrophy did not depend on PEG dose, but there was a significant relationship between the grade of lipodystrophy and escape from PEG, defined as loss of biochemical control in a patient who was previously controlled (*p* = 0.019). This observation suggests that the presence of lipodystrophy might influence the response to treatment. The female prevalence, observed also in previous studies [103–106], might be due to a greater accumulation of subcutaneous adipose tissue or to a gender-specific adipocyte response. Only few patients discontinued treatment due to lipodystrophy. Generally, lipohypertrophy regressed in all patients after medication was discontinued.

Overall, in patients receiving PEG, injection sites should be monitored for early signs of lipodystrophy and patients should be trained to frequently change the site of injection.

### Reproductive system and pregnancy

In women with acromegaly, reproductive disorders, including menstrual abnormalities, galactorrhoea, and decreased libido, are commonly reported, occurring in 40–80% of patients [107–110]. In particular, women with acromegaly often present with menstrual irregularity, mainly oligo-amenorrhea, associated with anovularity and infertility. Hypopituitarism or a direct action of GH and IGF-I excess on the pituitary–gonadal axis or the co-existence of hyperprolactinemia due to mass effect or to prolactin hypersecretion by the tumor have been proposed as potential mechanisms for the impairment in gonadotropin secretion, leading to ovarian dysfunction and consequent infertility [108–110]. Moreover, IGF-I excess was found to be associated with polycystic ovarian syndrome (PCOS) or a PCOS-like phenotype in 50% of acromegalic women with newly diagnosed or uncontrolled disease, despite previous treatment with surgery or SRL, particularly in those with small tumors and intact gonadotropinaxis [111]. However, few data are to date available about the fertility outcome in acromegalic women [112–117], since approximately 100 pregnancies have been described in literature [112]. Consequently, the management of acromegaly during pregnancy has to be precisely defined and the benefit-to-risk profile of the different treatment options need to be evaluated at the single patient level due to the limited data available on their safety and efficacy.

Generally, in patients with GH-secreting pituitary microadenomas or small tumor remnants, medical therapy can be safely withdrawn shortly after pregnancy confirmation [112, 117], carefully monitoring clinical symptoms of potential tumor enlargement (visual field); whereas in those with macroadenomas at high risk of tumor growth, medical therapy can be maintained throughout the pregnancy [112, 117]. Dopamine agonists (DA) and SRL represent the treatment of choice in acromegalic women during pregnancy, since the clinical cases reported so far have demonstrated that the use of these drugs in pregnant patients did not affect pregnancy course and fetal development [112, 113, 116, 118, 119].

The safety of PEG in pregnancy, as well as its potential fetal effects, is yet to be assessed. Early embryonic development and teratology studies in pregnant rabbits have failed to demonstrate any evidence of teratogenic effects at subcutaneous doses of 1, 3, and 10 mg/kg/day. At the dose of 10 mg/kg/day, a slight and reproducible increase in post-implantation loss was observed. However, this dose is ten times the maximum therapeutic dose, based on body surface area [120]. Moreover, it has to be considered that animal studies are not always predictive of human responses to pharmaceutical agents [120]. Nevertheless, PEG cannot be recommended in human pregnancies. So far, only two cases of women that conceived during PEG therapy were reported in detail [120, 121] and a recent analysis [122] identified 35 unique pregnancies from the Pfizer’s Global Safety Database including pregnancies during PEG exposure. Qureshi et al. [120] reported the first case in the literature of a 29-year-old woman with previously uncontrolled acromegaly, that conceived at her first cycle of in vitro fertilization and intra-cytoplasmic sperm injection, after IGF-I normalization during PEG monotherapy. PEG was immediately discontinued. Pregnancy was complicated by gestational diabetes and deterioration of visual field, but no tumor enlargement was observed. Conservative management with elective cesarean section was performed at 38 weeks of gestation and a healthy boy was delivered. Subsequently, Brian et al. [121] reported a case of a 26-year-old female with uncontrolled acromegaly, despite surgery, DA, and SRL therapy. After IGF-I normalization with PEG monotherapy, the patient conceived and continued PEG therapy throughout pregnancy. The PEG dose was increased during pregnancy up to 25 mg/daily. No complications were reported during pregnancy, an elective cesarean was performed at 40 weeks of gestation and a healthy girl was delivered. Interestingly, maternal and fetal PEG levels were also evaluated in this woman. Maternal PEG levels were consistent with a 25-mg daily dosage, whereas fetal PEG levels were minimal and near the range detected in untreated acromegalic patients. The GH variant levels in maternal and cord blood were within the normal ranges, and PEG levels in breast milk were below the lower limit of quantification of the assay and similar to those found with the same assay in milk samples from normal mothers.

Van der Lely et al. [122] recently reported the largest series of data available to date, which seem not to suggest adverse consequences of PEG on pregnancy outcome in acromegaly. In particular, they reviewed data on 35 pregnancy outcomes of acromegalic patients exposed to PEG included in the Pfizer’s Global Safety Database. Of these 35 pregnancies, 19 were included in the ACROSTUDY. Twenty-seven out of 35 pregnancy cases involved maternal exposure to PEG and 8 cases involved paternal exposure to PEG. A normal newborn was reported in 14 cases; while in 4 cases, a live birth was reported, but the outcome of the birth was not specified. In nine cases, the fetal outcome was not reported. In three cases of maternal exposure that reported a normal newborn, PEG was continued throughout the pregnancy at a mean dose of 12.1 mg/day. In two cases a non-PEG-related spontaneous abortion occurred, in three cases a cesarean section was performed, and in one case a non-PEG-related ectopic pregnancy occurred and in three women gestational diabetes developed.

In summary, until now, the available data do not suggest adverse effects on pregnancy outcome associated with PEG therapy, although the number of pregnancies during exposure to PEG reported in the literature is too small to issue any types of indication. For this reason, the use of PEG during pregnancy is not recommended unless absolutely clinical necessary (i.e., severe acromegaly-related diabetes, or cardio-respiratory complications in SRL-resistant patients). The potential fetal effects of PEG are still unknown. In particular, more studies are needed to evaluate PEG effect on fetal growth and possible interference on the placental GH activity, since during pregnancy placenta produces human GH, that differs from pituitary GH by 13 amino acids [123] and displays its biological effect through the same GH receptor [124]. PEG therapy during breast-feeding should also be avoided as it is unclear whether this drug is excreted or not in breast milk.

## Resistance to SRL

PEG finds its main indication in patients partially or completely resistant to SRL treatment [29, 50]. Some clinical and pathological aspects have been proposed to be effective in predicting the response to SRL. In particular, a better response seems to be associated to female sex, older age, hypo-intensity in T2 at MRI, high SST2R expression, high AIP expression at immunohistochemistry, low Ki67 index, a “densely granulated” pattern at electronic microscopy [125, 126]. A partial to absent response to SRL is also related to particular genetic alterations as observed in patients bearing AIP gene mutations or the newly discovered X-linked acrogigantism [127].

To define the therapeutic efficacy of SRL treatment, the effects on both GH/IGF-1 secretion and tumor mass are considered, these parameters being generally evaluated after 6–12 months of therapy with both octreotide LAR and lanreotide ATG at “conventional” maximal doses (i.e., 30 mg/28 days and 120 mg/28 days, respectively) [128, 129]. Current guidelines define biochemical control as random GH values <1 ng/ml, IGF-I within the normal age-specific levels, and GH nadir following OGTT less than 1 or 0.4 ng/ml [1, 130].

However, some aspects of the definition of resistance to SRL need to be improved. In particular, the limitations of this definition so far are tied to the target GH value (1 vs 2.5 ng/ml), the clinical relevance of a significant tumor shrinkage, the use of percentage reductions of GH/IGF-I vs absolute values, the improvement of clinical signs and symptoms. It is worth noting that a relevant parameter to consider in defining a patient as resistant or partially responsive is the duration of SRL therapy [131]. This aspect turns out to be particularly important because the delay between the onset and the diagnosis of acromegaly has not been reduced in the last decades [132], and such a diagnostic delay requires rapid normalization of both GH and IGF-1 secretion to avoid further exposure to elevated GH/IGF-I concentrations.

### Management of SRL-resistant patients

A patient with GH/IGF-1 reduction <50% and/or tumor mass shrinkage <20–25% may be considered as SRL-resistant. In this respect, the results obtained after 6 months seems to be predictive of the long-term efficacy of SRL treatment [128]. In particular, it has been proposed that when a patient does not show any biochemical responses after 6 months of treatment it is advisable not to continue with SRL and to switch towards PEG in monotherapy. No studies are so far available showing the efficacy of both pasireotide and SRL at high doses in improving the biochemical response in SRL-resistant patients.

In patients bearing pituitary macroadenomas, it has been proposed to prolong SRL treatment up to 12–18 months to evaluate a possible late effect on tumor mass independently from the efficacy on hormone secretion. In particular, in these patients, a combined SRL–PEG treatment might be proposed to normalize GH/IGF-1 secretion waiting for a possible delayed SRL effect on tumor mass.

### Management of patients with a partial biochemical response to SRL

A GH/IGF-I reduction >50% without reaching normalization is considered according to recent guidelines as a partial biochemical response to SRL [51]. In patients with partial response to conventional maximal doses of SRL, different medical options can be considered.

PEG in monotherapy was shown to effectively control IGF-I secretion in the large majority of these subjects [29], being also often effective in normalizing glucose profile. A recently published study [20] showed that SRL at higher doses (i.e., octreotide LAR 60 mg/28 days) were effective in normalizing hormone secretion in about 36% of acromegalic patients. In a similar setting, pasireotide reduced mean GH levels to less than 2.5 ng/mL in 35–45% of cases and normalized IGF-I in 15–20% [50]. However, while high doses of SRL did not affect glucose metabolism [70], pasireotide induced a negative effect on glucose metabolism.

#### Timing of the switch from conventional SRL

Although 12 months of treatment are traditionally necessary to define a partial resistance to SRL [128], it is conceivable to switch to a different option within 6 months from the beginning of SRL if the GH/IGF-I levels remain, in absolute terms, well above the target values.

#### Type of switch

ually the achievement of a complete biochemical control. However, in a “patient-oriented” strategy, other elements should be considered in the therapeutic decision-making, since they may dictate the necessity for a rapid biochemical correction of the disease or the preference for one or the other medical tool or their combination. In particular, the absolute values of GH and IGF-I as well as the presence and severity of comorbidities and of tumor residues having a significant mass effect, therapy costs, and patient compliance should be taken into account when deciding to switch to a different therapy.

PEG represents the first choice therapy in patients in whom the severity of the disease requires a quick IGF-1 normalization, through appropriate titration of the drug [27, 45], as well as in not fully compensated diabetic patients. The latest guidelines recognize the relevance of PEG treatment either in monotherapy or in combination with SRL because it is highly effective in IGF-I normalization [29, 51]. PEG in monotherapy was proven to be effective in normalizing IGF-I in up to 88% of patients resistant to SRL [9]. The combination therapy with SRL appears an effective option in those patients in whom SRL had shown to be effective in reducing pituitary tumor mass.

High doses of SRL [20] might be used when the clinical situation does not require an immediate normalization of GH/IGF-1 secretion, but the percentages of normalization are lower than those observed with PEG. In this subset of patients, pasireotide might be an alternative option, though high-dose SRL should be preferred in those with diabetes mellitus.

## Conclusions

This review focuses on some emerging aspects of the management of acromegaly with particular reference to their impact on the clinical use of PEG which may be considered still suboptimal. In fact, PEG remains the most powerful tool to control IGF-I in acromegaly and therefore the medical treatment through which we can obtain the most important clinical effects either in terms of signs and symptoms or of quality of life or of comorbidities. The goal for the clinician is to offer this treatment option to all the patients that are likely to benefit from it and to adequately monitor its clinical and biochemical effects. From the review of the most current literature, it appears that the number of patients with acromegaly exposed to PEG worldwide has become quite elevated and their prolonged follow-up allows us to deal quite satisfactorily with many clinical issues, including most importantly the safety of the treatment. In particular, this review highlights that issues concerning the possible tumor (re)growth under PEG have been significantly down-played by the recent literature although in some patients at high risk in this regard the co-treatment with SRL can be considered to further minimize the risk. On the other hand, the positive or neutral impact of PEG on glucose metabolism has been highlighted as it may indicate PEG as the best medical option in acromegaly patients with diabetes mellitus. We have also reviewed some aspects of the treatment with PEG which are less frequently dealt with, also due to the limited experience available, such as sleep apnoea and pregnancy. In this latter case, although the very limited published material is reassuring, PEG use remains not indicated in acromegaly patients undergoing pregnancy. Finally, in an attempt to adequately define all the patients to whom PEG option may be offered, we dealt with the current definition of SRL resistance. This issue so far has been mainly defined based on biochemical and tumor elements; however, we propose to consider other aspects that can refine the definition, taking into account some literature trends which try to predict response (or no response) to SRL and elements that may affect the necessity for the clinician to obtain a rapid and full response to treatment. In conclusion, PEG increasingly appears to be an effective and safe medical option for many patients not controlled by SRL but its use still needs to be optimized.
